# Effectiveness of early intervention and combination treatment with monoclonal antibodies and antivirals in oncohematological patients with SARS-CoV-2: a retrospective experience

**DOI:** 10.3389/fimmu.2025.1524525

**Published:** 2025-03-28

**Authors:** Silvia Di Bari, Francesco Izzo, Livia Bresciani, Giulia Mancarella, Silvia Garattini, Andrea Gasperin, Daniela Di Trento, Alessandra Grimaldi, Alberico Parente, Raffaella Marocco, Anna Carraro, Blerta Kertusha, Tiziana Tieghi, Cosmo Del Borgo, Serena Vita, Mariasilvia Guardiani, Caterina Pasquazzi, Alessandra Spagnoli, Danilo Alunni Fegatelli, Miriam Lichtner

**Affiliations:** ^1^ Infectious Disease Unit, Sant’Andrea Hospital, Sapienza University of Rome, Rome, Italy; ^2^ Infectious Disease Unit, Santa Maria Goretti Hospital, Sapienza University of Rome, Latina, Italy; ^3^ National Institute for Infectious Diseases Lazzaro Spallanzani, Rome, Italy; ^4^ Department of Public Health and Infectious Diseases, Sapienza University of Rome, Rome, Italy; ^5^ Department of Neuroscience, Mental Health, and Sense Organs, NESMOS, Sapienza University of Rome, Rome, Italy

**Keywords:** SARS-CoV-2, oncohematological patients, combination therapy, COVID-19 pneumonia, antivirals, monoclonal antibodies

## Abstract

Patients with acute SARS-CoV-2 and pre-existing oncohematological conditions challenge clinicians due to a heightened risk for severe COVID-19 and forced deferral of cancer treatment. Different treatment approaches aim to either prevent the progression of mild disease (“early therapy”) or to treat more severe COVID-19. Currently, there is limited evidence supporting the effectiveness of a tailored approach for oncohematological patients. We present a real-world experience from two university hospitals. In this retrospective study we recruited oncohematological patients hospitalized for SARS-CoV-2 pneumonia between March 2020 and June 2023 from two hospitals in Latium, Italy. Patients with COVID-19 pneumonia received either antiviral or monoclonal antibodies (MoAb) alone, a dual therapy (antiviral with MoAb) or a triple therapy (two different antivirals and MoAb). The study aimed to evaluate the practical management of hospitalized oncohematological patients with COVID-19. We focused on the impact in patients with COVID-19 related pneumonia of specific therapies, early treatment, and tixagevimab-cilgavimab prophylaxis on in-hospital mortality and viral clearance time. Overall, 101 patients were recruited, 76 (75.24%) patients developed pneumonia, and 16 (15.84%) patients died from any cause. While most patients (75,25%) did not receive “early therapy”, those who did had a higher chance of survival (p=0.04). Furthermore, the pneumonia subgroup treated with early therapy demonstrated a higher survival rate as well (p=0.02). Out of the hospitalized patients triple therapy resulted in lower mortality (all patients survive in this group). This group also showed a significant reduction in the time to viral clearance from the first day of the evaluated therapy (6 days [IQR 4;9]), compared to patients treated with only remdesivir (17 days [IQR 8;37]) (p=0.03). Our findings demonstrate that early therapy significantly reduces in-hospital mortality, while triple therapy accelerates viral clearance in hospitalized patients. These results, in line with recent studies, underscore the critical importance of prompt treatment and a multitargeted pharmacological approach for optimizing outcomes in oncohematological patients with SARS-CoV-2. Future research, involving larger cohorts, should delve deeper into COVID-19 treatment strategies for this vulnerable population, with a particular emphasis on the elderly, who continue to experience high mortality rates.

## 1 Introduction

The virus identified as severe acute respiratory syndrome coronavirus 2 (SARS-CoV-2) is responsible for the potentially fatal Coronavirus Disease 19 (COVID-19). As of October 2024, there have been 777,368,929 confirmed cases of COVID-19 worldwide, with 7,087,731, confirmed deaths reported (1). Owing to the bolstered herd and individual immunity arising from both vaccination (2, 3) and prior exposure to the infection (4), along with a notable reduction in morbidity and mortality linked to the more recent circulating variants (5), a substantial segment of the population is now safeguarded from severe repercussions of the disease. Nonetheless, certain demographic subgroups continue to face elevated risks. One such group of patients is those affected by hematological malignancies. These diseases can directly induce myelosuppression and lymphosuppression due to the replication of neoplastic cells outcompeting leukocyte population. Maia et al. demonstrated that this population has lower levels of monocytes, natural killer cells, T lymphocytes, and B lymphocytes when compared with other patients with SARS-CoV-2 infection (6). Moreover, treatments against tumor cells often have a cytotoxic effect on healthy cells, resulting in the destruction of immunocompetent cells in the immune system. Rituximab targets B cells by binding to the CD-20 receptor, leading to a reduction in the ability to produce specific neutralizing immunoglobulins against SARS-CoV-2 after vaccination or exposure to the virus (7). Additionally, this specific subset of patients is especially prone to severe and prolonged infections (8) that can result in the selection of significant viral variants (9, 10). Moreover, considering all of this, clinicians often decide to defer hematological treatment, increasing mortality (11).

Until recently, few clinical studies had been conducted to assess the optimal therapeutic approach to improve poor outcomes and reduce time to viral clearance in oncohematological patients. Moreover, most existing studies were mostly retrospective, involved few participants, or were case series. In a Polish study the intravenous antiviral (AV) remdesivir, administered to hospitalized patients, was able to reduce mortality in patients with malignancies (12); however, only a minority of patients in this study had hematological malignancies. In other studies, such as the retrospective study conducted by Alexandra Martin-Onraët, remdesivir did not reduce the risk of disease progression in hospitalized patients (13). In any case, in the absence of a sufficient humoral immune response, AV therapy alone may not be sufficient to achieve viral clearance (14).

EPICOVIDEHA, a European electronic registry for oncohematological patients with SARS-CoV-2 infection, has finally enabled studies with larger patient samples (15). This platform demonstrated the effectiveness of sotrovimab and tixagevimab-cilgavimab in reducing mortality in patients with critical COVID-19 (HR 0.13) (16). A retrospective study conducted by the Italian Adult Hematologic Diseases Group (GIMEMA) showed that MoAb therapy in oncohematological patients with mildly symptomatic COVID-19 reduced the median time to viral clearance (17). Furthermore, MoAb therapy has proven effective in reducing the time to viral clearance, the duration of hospitalization, and the need for intensive care compared to the control group of patients with COVID-19 before such therapy was available (18).

Given the fragility of oncohematological patients compared to the general population, it may be necessary to combine therapies with an inhibitory effect on viral proliferation (antivirals), with drugs capable of inducing a passive immune response, such as MoAb. Ideally, such therapies should not only prevent disease progression but also shorten the duration of the SARS-CoV-2 infection, as detected by molecular and antigenic tests.

The simultaneous use of AV therapies with different mechanisms of action is supported by the increased effectiveness of combination therapy in other infections caused by RNA viruses. For example, drug combinations with different mechanisms are already used in the treatment of HIV or HCV infections. For this reason, with SARS-CoV-2 infections, the option of using AVs with different mechanisms, such as remdesivir (with polymerase activity) and Nirmatrelvir/ritonavir (with protease activity) has been considered. Furthermore, studies have shown *in vitro* synergistic action of nirmatrelvir and molnupiravir towards both wild-type and Omicron variants (19, 20). Regarding combination therapy for oncohematologic patients, past literature has mostly consisted of case studies or case series (21–24). A recent case series demonstrated that the combination of two antivirals (remdesivir and nirmatrelvir-ritonavir) is a promising treatment option for persistent COVID-19 in immunocompromized patients with B-cell line hematologic malignancies and/or those receiving anti-CD20 therapies (25). Moreover, a recent study showed that triple therapy is associated with high rates of clinical and virologic clearance in oncohematologic patients (26). To enhance knowledge regarding the effect of combination therapy (dual and triple therapy) on outcomes and viral clearance, we conducted this study.

Certain MoAbs have been shown to effectively prevent disease progression in this fragile patient population. Indeed, in the Italian GIMEMA study the mortality rate among patients treated with different MoAb was only 3.3%, much lower than expected for oncohematological patients (17). Early therapy with oral AVs targeting SARS-CoV-2 have also been tested in hematological patients. Another Italian multicenter study showed how oral AVs frequently failed to prevent severe disease in hematological patients with a COVID-19. Indeed, in this group COVID-related mortality remained relatively high at 6.1% (27). Conversely, a recent study demonstrated that early combination treatment in immunocompromised patients prevents prolonged viral shedding (28). Moreover, another study demonstrated that the use of combination treatment with two antivirals and sotrovimab in the early phase of COVID-19 in immunocompromised leads to both clinical and virological responses (29). These findings highlight the need for combination therapy, even in outpatient settings. Given the conflicting results in the literature, we conducted this study to better assess early therapy effect on mortality and viral clearance.

## 2 Materials and methods

### 2.1 Study endpoint and design

In this multicenter retrospective observational study, we collected data from consecutively hospitalized oncohematological patients at two Italian hospitals Santa Maria Goretti Hospital in Latina and Sant’Andrea Hospital in Rome. We included patients admitted between July 2020 and June 2023 regardless of whether SARS-CoV-2 was the primary cause for hospitalization. The inclusion criteria were I) diagnosis of COVID-19 confirmed by antigen testing or molecular nasopharyngeal swab (NPS) for SARS-CoV-2; II) hospitalization; III) age > 18 years old; IV) onco-hematological conditions.

The aim of the study was to evaluate and describe the practical approach to managing oncohematological patients hospitalized with COVID-19. The primary endpoint was to assess the impact of specific COVID-19 therapies (including rescue therapy), early therapy, and prophylaxis with tixagevimab-cilgavimab on in-hospital mortality. The secondary endpoint was to evaluate the time to SARS-CoV-2 viral clearance under different therapeutic strategies, calculated from both the first positive test for SARS-CoV-2 and the initiation of specific COVID-19 therapy.

Statistical analysis was performed on the entire study population and on patients with COVID-19 pneumonia.

To assess the effectiveness of different therapeutic options, pneumonia patients who received targeted anti-SARS-CoV-2 therapy were divided into four groups based on the treatment administered:

- Monotherapy with MoAb;
- Monotherapy with remdesivir;
- Combination therapy with remdesivir plus MoAb;
- Triple therapy (remdesivir plus MoAb plus oral AV drugs).

Viral clearance for each patient was assessed via two consecutive antigenic swabs. To allow a more specific comparison between therapy groups, the time to viral clearance was calculated also from the start date of the specific therapy being studied.

To assess the impact of different therapeutic choices on the time to viral clearance, the latter was categorized into three groups: within 14 days, between 14 and 30 days and beyond 30 days. It was recorded whether the patient had undergone tixagevimab/cilgavimab prophylaxis and, if so, whether it had been administered within six months of the SARS-CoV-2 infection. Early therapy with AVs or MoAb was documented, specifying the type of MoAb used.

Age, vaccination history and specifics regarding the patient’s oncohematological condition were collected. Oncohematologic disease categories were: Hodgkin lymphoma (HL), non-Hodgkin lymphoma (NHL), chronic myelogenous leukemia (CML), chronic lymphocytic leukemia (CLL), acute myelogenous leukemia (AML), multiple myeloma (MM), myelofibrosis (MF), and myelodysplastic syndrome (MDSs). The specific treatment administered for the neoplastic condition and its timing in relation to the SARS-CoV-2 infection (within one year of infection) were also recorded.

Clinical and radiographic data were collected and de-identified before being stored in a digital database. Outcomes were categorized as follows: deceased due to COVID, deceased due for reasons unrelated to COVID, and discharged. A death was attributed to COVID-19 if the patient remained positive for SARS-CoV-2 at the time of death, had severe COVID-19 at the time of death, or died in the absence of other potential causes of death. The choice between various anti-SARS-CoV-2 therapies, including MoAb (bamlativimab-etesevimab; casirivimab-imdevimab; sotrovimab), remdesivir, and oral AVs (nirmaltrevir-ritonavir and molnupinavir), was based on the national guidelines at the time of infection, the recommendations of the Italian Medicines Agency (AIFA), the decisions of the infectious disease specialist and hematologist assigned to the case, the availability of the drugs in the hospital pharmacy and the circulating dominant SARS-CoV-2 variant at the time of treatment.

The decision to use corticosteroids and immunotherapy with tocilizumab was based on clinical practice and guidelines, with off-label use for the combination therapy.

COVID-19 pneumonia, documented radiologically, was categorized as mild, moderate, or severe, based on the Berlin criteria for acute respiratory distress syndrome (30):

- Mild: 200 mmHg < PaO2/FiO2 ≤ 300 mmHg* with positive end-expiratory pressure or continuous positive airway pressure ≥ 5 cm-H2O;
- Moderate: 100 mmHg < PaO2/FiO2 ≤ 200 mmHg with positive end-expiratory pressure ≥ 5 cm-H2O;
- Severe: PaO2/FiO2 ≤ 100 mmHg with positive end-expiratory pressure ≥ 5 cm-H2O.

### 2.2 Statistical analysis

Data were expressed as mean and standard deviation (SD) or median and interquartile range (IQR) for continuous variables, based on their distribution, and as counts and percentages for categorical variables. Comparisons between treatment groups were performed using the chi-squared test, Student’s t-test or Mann-Whitney U test, as appropriate.

For continuous data, we used the t-test (Student’s t-test) when the samples were (approximately) normally distributed, which was tested using the Shapiro-Wilk test. When the normality assumption was not met, we used the Mann-Whitney U test as a non-parametric alternative for two-group comparisons. For categorical data divided into two groups, we used the chi-square test when expected cell counts were sufficiently large (≥5) and Fisher exact test otherwise. For comparisons involving three or more independent groups, since normality was not guaranteed by the Shapiro-Wilk test, we used the Kruskal-Wallis test as a non-parametric alternative to one-way ANOVA.

Finally, for survival analysis, we used the log-rank test under the assumption of independence of survival times and proportional hazards. The primary outcome was overall survival rate (in-hospital mortality). The secondary outcome was the time to viral clearance, defined as the time from the first positive swab to negative swab or death due to any cause. Participants without documented death at the time of analysis were censored at the date of last known contact. The estimate of the overall survival was obtained from the Kaplan-Meier method and displayed graphically. Differences in progression-free survival (PFS) between treatment arms were analyzed by the log rank test.

To evaluate the effectiveness of the four anti-SARS-CoV-2 therapy groups, global time to viral clearance, calculated from the first positive swab, was considered as a categorical variable with the following range: within 14 days, between 14 to 30 days, and greater than 30 days.

Moreover, to further analyze the impact of the specific anti-SARS-CoV-2 therapy in patients with prolonged infection, the negativization time was also defined from the beginning of the therapy.

To address the issue of multiple testing corrections in the analysis of therapy groups, the Wilcoxon test with a Bonferroni correction was applied. This decision was made to mitigate the effects of multiple comparisons and ensure robust and reliable results. The paired Wilcoxon test was used to compare differences within the paired groups, while the Bonferroni correction was applied to reduce the risk of Type I errors. This approach allowed us to maintain adequate control over the overall significance level, ensuring the statistical integrity of our analysis.

The data were analyzed using the open-source software R, version 4.3.1, and GraphPad Prism 8.0. The significance level for all analyses was set at 0.05. This study was approved by the Ethics Committee Lazio 2 (protocol number 0038491/2022), as established by the Ministry of Health of the Italian Government. Each subject provided written informed consent for data collection and the specific anti-SARS-CoV-2 treatment.

## 3 Results

**Figure 1:**
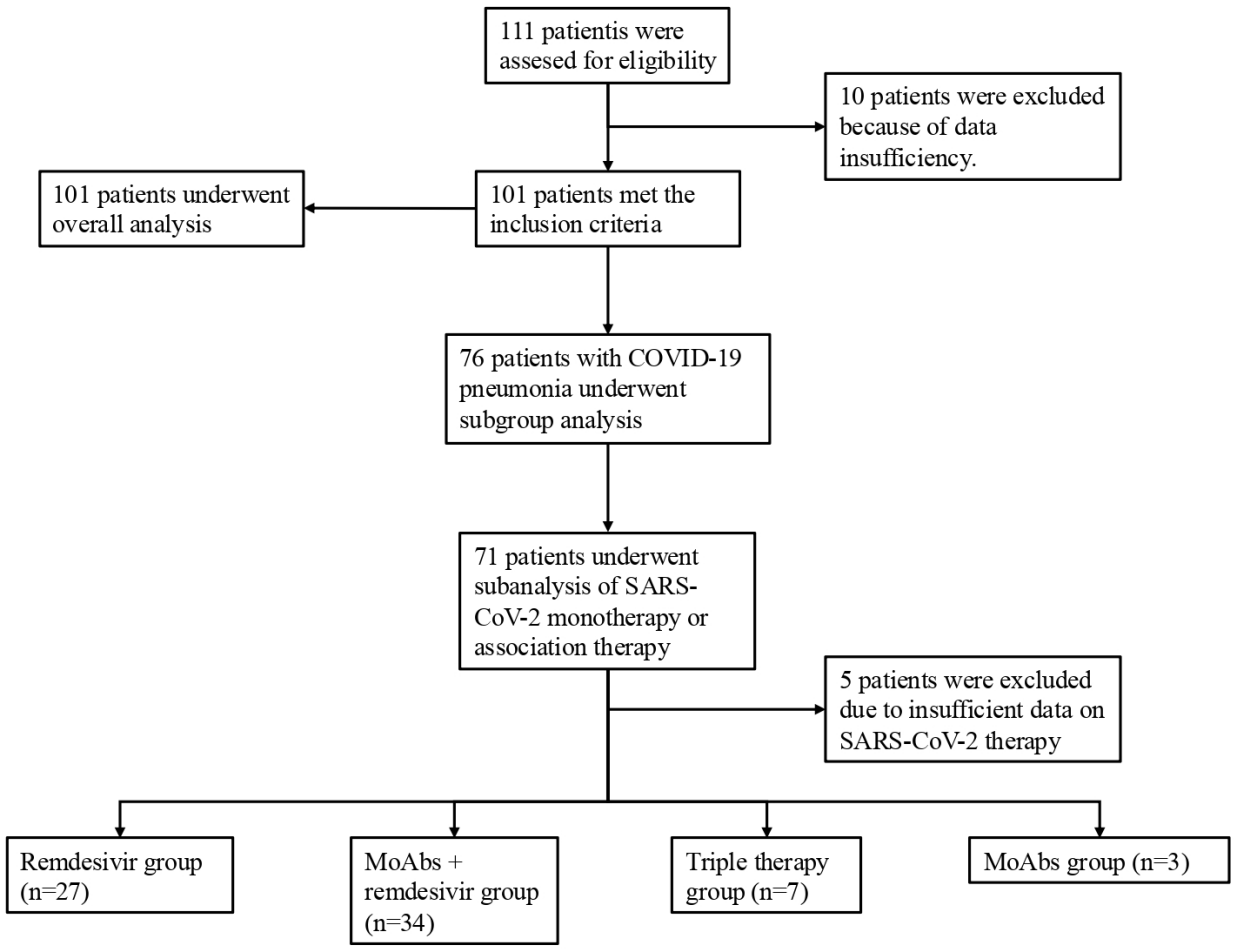
Flowchart of the study.

In this study, 101 onco-hematologic patients hospitalized with SARS-CoV-2 infection were enrolled at the infectious disease unit of Santa Maria Goretti Hospital in Latina and the Sant’Andrea University Hospital in Rome between July 2020 and June 2023. The flow chart of our study is presented in **Figure 1**. The average age was 71.02 ± 11.4 years. 37.62% of the study population consisted in females. Among the participants, 76 had radiologically confirmed interstitial pneumonia through chest computed tomography: 42 cases were mild, 25 moderate, and 9 severe. Among these patients, 73.68% required supportive oxygen therapy, while 25% needed high-flow oxygen therapy or CPAP. The majority of patients had cardiovascular diseases (57.43%), 23.76% had diabetes, 22.77% had chronic respiratory diseases, and13.86% had kidney diseases. Additionally, 75.25% of patients were over 65 years old. Hematologic disorders were categorized as follows: Hodgkin’s lymphoma (4.95%), non-Hodgkin’s lymphoma (43.56%), acute myeloid leukemia (8.91%), chronic myeloid leukemia (3.96%), chronic lymphocytic leukemia (21.78%), multiple myeloma (13.86%), myelofibrosis (0.99%), and myelodysplasia (4.95%). Hematologic therapy was stratified into the following therapeutic categories:

- Chemotherapy alone (28.71%);
- Ibrutinib (8.91%);
- Treatment regimen including anti-CD20 (18.81%);
- Stem cell transplantation (5.94%);
- Treatment regimen including anti-Bcl-2 (2.97%);
- IMiDs (Thalidomide immunomodulatory analogs) +/- protease inhibitor (7.92%);
- Chimeric Antigen Receptor T-cell therapy (CAR-T) (0.99%);
- No therapy (25.74%).

More specifically, 19 patients underwent treatment with anti-CD20, of these, 15 were treated with rituximab and 4 with obinutuzumab. Sixty-five percent of patients underwent specific hematologic therapy within one year of infection.

The majority of subjects (84.16%) were vaccinated. Among them, only one patient received a single vaccine dose, 15.84% completed the standard vaccination cycle (at least two doses), 51.49% received a booster dose (three doses), and 15.84% received four doses. The remaining 15.84% of patients did not receive the SARS-CoV-2 vaccination. All participants in the study were oncohematologic patients classified as immunosuppressed. Consequently, they were vaccinated with an mRNA vaccine rather than a viral vector vaccine.

The median time to viral clearance in the entire examined population was 21 [13; 34] IQR, while considering the time to viral clearance from the SARS-CoV-2 therapy under study, the median was 13 [6; 27]. The difference between the onset of COVID-19 symptoms and the administration of the therapy was 6 days [IQR: 3; 15.75], and the median of hospital stay was 15 days [IQR 10; 29].

Only sixteen patients (15.85% of overall population) had previously received tixagevimab- cilgavimab as prophylaxis, and among them, 71.43% contracted SARS-CoV-2 infection within six months of MoAb administration. The majority of patients (n pt, 75.2%) did not receive early therapy. Among those who did (24.75%), early therapy treatment included MoAb (n=8), remdesivir for 3 days (n=7), nirmatrelvir-ritonavir (n=6), or molnupiravir (n=4). Overall mortality was 15.84% (16 patients), with 9 death (56.25%) attributed to COVID-19-related causes and 7 (43.75%) to non-COVID-19-related causes. We analyzed the differences in patient characteristics and treatments categorized by outcome, as shown in **Table 1**.

**Table 1:** Clinical characteristics of overall cohort, characterized by outcome.

Characteristic	All patients n (%) = 101 (100)	Discharged n (%) = 85 (84.15)	Death n (%) = 16 (15.8)	P-value
Sex, female, n (%)	38 (37.62)	32 (84.21)	6 (15.79)	>0.9
Age at hospitalization (years)				**0.008**
Mean (SD)	71.02 (11.40)	69.75 (11.74)	77.75 (6.14)	
Median [25%-75%]	72 [65-79]	70 [63-79]	77 [74-81]	
Type of therapy				0.9
MoAb, n (%)	9 (9.68)	8 (88.89)	1 (11.11)	
Remdesivir, n (%)	34 (36.56)	28 (82.35)	6 (17.65)	
Remdesivir +MoAb, n (%)	43 (46.24)	35 (81.39)	8 (18.60)	
Triple, n (%)	7 (7.53)	7 (100)	0 (0.00)	
Pneumonia	76 (75.25)	64 (84.21)	12 (15.78)	>0.9
Length of hospital stay (days)				**0.036**
Mean (SD)	21.72 (18.33)	19.19 (13.29)	35.19 (31.94)	
Median [25%-75%]	15 [10-29]	14 [10-27]	29 [15-46]	
Time to viral clearance (days)				0.2
Mean (SD)	28.05 (21.74)	27.54 (21.64)	38.50 (24.31)	
Median [25%-75%]	21 [13-34]	21 [12-33]	32[27-44]	
Difference between the start of therapy and first negative swab (days)				
Mean (SD)	19.57 (18.93)	17.99 (16.45)	28 (28.02)	0.14
Median [25%-75%]	13 [6-27]	13 [6-26]	22 [11-37]	
Hematological malignancy				
HL, n (%)	5 (4.95)	5 (100)	0 (0.00)	>0.9
NHL, n (%)	44 (43.56)	37 (84.1)	7 (15.9)	>0.9
AML, n (%)	9 (8.91)	9 (100)	0 (0.00)	0.3
CML, n (%)	4 (3.96)	3 (75)	1 (25)	0.5
CLL, n (%)	22 (21.78)	17 (77.27)	5 (22.73)	0.3
MM, n (%)	14 (13.86)	13 (92.86)	1 (7.14)	0.5
MF, n (%)	1 (0.99)	0 (0.00)	1 (100)	0.2
MDSs, n (%)	5 (4.95)	4 (80)	1 (20)	0.6
Comorbidity				
Age>65 years old, n (%)	76 (75.25)	60 (78.94)	16 (21.05)	**0.010**
Cardiovascular disease, n (%)	58 (57.43)	48 (82.75)	10 (17.24)	0.7
Diabetes, n (%)	24 (23.76)	18 (75.00)	6 (25.00)	0.2
Respiratory diseases, n (%)	23 (22.77)	16 (69.56)	7 (30.43)	**0.047**
Nephropathy, n (%)	14 (13.86)	11 (78.57)	3 (21.43)	0.7
Infection within six months of full vaccination, n (%)	24 (35.29)	18 (75)	6 (25)	0.3
Vaccination				>0.9
0, n (%)	16 (15.84)	13 (81.25)	3 (18.75)	
1 dose, n (%)	1 (0.99)	1 (100)	0 (0.00)	
2 doses, n (%)	16 (15.84)	14 (87.50)	2 (12.50)	
3 doses, n (%)	52 (51.49)	43 (82.69)	9 (17.31)	
4 doses, n (%)	16(15.84)	14 (87.50)	2 (12.50)	
tixagevimab/cilgavimab prophylaxis, n (%)	16 (15.84)	14 (87.5)	2 (12.50)	>0.9
Infection within six months of prophylaxis with tixagevimab/cilgavimab, n (%)	10 (9.90)	8 (80)	2 (2)	>0.9
Early therapy, n (%)	25 (24.75)	24 (96)	1 (4)	0.11
CRP (mg/dL with normal values <0.5 mg/dL)				0.6
Mean (SD)	6.75 (8.00)	9.61 (7.94)	10.48 (8.51)	
Median [25%-75%]	7.99 [3.73-13.19]	7.81 [3.07-13.39]	8.51 [5.69-12.73]	
Hematologic therapy				0.9
Chemotherapy alone, n (%)	29 (28.71)	24 (82.75)	5 (17.25)	
Ibrutinib, n (%)	9 (8.91)	6 (66.67)	3 (33.33)	
Including anti-CD20 such as:	19 (18.81)	17 (89.47)	2 (10.53)	
Rituximab, n (%)	15 (78.9)	13 (86.7)	2 (13.3)	
Obinutuzumab, n (%)	4 (2.1)	4 (100)	0 (0)	
Stem cell transplantation, n (%)	6 (5.94)	5 (83.33)	1 (16.67)	
Including anti-Bcl-2, n (%)	3 (2.97)	3 (100)	0 (0.00)	
IMiDs +/- protease inhibitor, n (%)	8 (7.92)	7 (87.5)	1 (12.5)	
CAR-T, n (%)	1 (0.99)	1 (100)	0 (0.00)	
No therapy, n (%)	26 (25.74)	21 (84.00)	4(16.00)	
Infection within one year of hematologic infection, n (%)	65 (62.5)	54(83.1)	11(16.9)	>0.9

MoAb, monoclonal antibodies; Triple, triple therapy (two antivirals + one MoAb); HL, Hodgkin lymphoma; NHL, non-Hodgkin lymphoma; CML, chronic myelogenous leukemia; CLL, chronic lymphocytic leukemia; AML, acute myelogenous leukemia; MM, multiple myeloma; MF, myelofibrosis; MDSs, myelodysplastic syndrome, CRP, C-reactive protein; IMiDs, Thalidomide immunomodulatory analogs; CAR-T, Chimeric Antigen Receptor T-cell therapy. Bold values in the tables indicate statistically significant values.

Our analysis revealed that patients with an unfavorable outcome were older (p=0.008), more likely to be over 65 (p=0.010) and often had an underlying respiratory disease (p=0.047). Patients who died had longer hospital stays (p=0.036). Moreover, the severity of pneumonia was associated with an unfavorable outcome (p=<0.001). No other characteristics were significantly different between the two groups, even if only one patient on 25 (6%) treated with early therapy died, suggesting a protective effect. Moreover, 64.5% of pneumonia patients did not receive either tixagevimab/cilgavimab prophylaxis or early therapy. Notably, none of the nine patients who died due to COVID-19-related causes had received prophylaxis with tixagevimab/cilgavimab and/or early therapy. However, there was no statistically significant difference in mortality between these groups. For each patient, the C-reactive protein levels at admission were measured assessed in mg/dL with normal values <0.5 mg/dL; the mean was 9.75 ± 7.99, no correlation was found with respect to the outcome. Hematologic therapy did not have a significant impact on the outcome. A sensitivity analysis on the population with pneumonia categorized by outcome showed similar results, as illustrated in **Table 2**.

**Table 2:** Clinical characteristics of COVID-19 pneumonia population, characterized by outcome.

Characteristic	Patients with pneumonia n (%)= 76 (100)	Discharged n (%) = 64 (84.2)	Death n (%) = 12 (15.8)	P-value
Sex, female, n (%)	31 (40.79)	25 (80.64)	6 (19.35)	0.5
Age of hospitalization (years)				**0.021**
Mean (SD)	71.22 (10.95)	70.05 (11.30)	77.50 (5.92)	
Median [25%-75%]	72.50 [66.75-79.25]	70 [63.75-77.25]	78.50 [72.75-81.25]	
Type of therapy				0.7
MoAb, n (%)	3 (4.23)	3 (100)	0 (0)	
Remdesivir, n (%)	27 (38.03)	22 (81.48)	5 (18.52)	
Remdesivir +MoAb, n (%)	34 (47.89)	27 (79.41)	7 (20.59)	
Triple, n (%)	7 (9.86)	7 (100)	0 (0)	
Length of hospital stay (days)				0.12
Mean (SD)	20 (12.22)	18.69 (10.63)	27 (17.55)	
Median [25%-75%]	14.50 [10.75-28.25]	14 [10-27]	20.50 [14.75-39]	
Duration of viral clearance (days)				0.4
Mean (SD)	29.65 (23.49)	29.15 (23.34)	40 (29.55)	
Median [25%-75%]	22 [14-37]	22 [13.25-36.25]	31 [23.50-52]	
Difference between start of therapy and negative swab (days)				0.2
Mean (SD)	17.74 (16.77)	17 (16.90)	21.67 (16.19)	
Median [25%-75%]	12.50 [6-25]	10.50 [5.75-22]	20.50 [11.50-28]	
Severity of pneumonia				**<0.001**
Mild, n (%)	42 (55.3)	39 (92.8)	3 (7.2)	
Moderate, n (%)	25 (32.9)	23 (92)	2 (8)	
Severe, n (%)	9 (11.8)	2 (22.2)	7 (77.8)	
Hematological malignancy				
HL, n (%)	3 (3.95)	3 (100)	0 (0)	>0.9
NHL, n (%)	35 (46.05)	31 (88.57)	4 (11.43)	0.3
AML, n (%)	5 (6.58)	5 (100)	0 (0)	>0.9
CML, n (%)	2 (2.63)	1 (50)	1 (50)	0.3
CLL, n (%)	20 (26.32)	15 (75)	5 (25)	0.3
MM, n (%)	9 (11.84)	9 (100)	0 (0)	0.3
MF, n (%)	1 (1.32)	0 (0)	1 (100)	0.2
MDSs, n (%)	3 (3.95)	2 (66.66)	1 (33.33)	0.4
Comorbidity				
Age>65 years old, n (%)	59 (77.63)	47 (79.66)	12 (20.33)	0.058
Cardiovascular disease, n (%)	45 (59.21)	37 (82.22)	8 (17.78)	0.8
Diabetes, n (%)	19 (25)	13 (68.42)	6 (31.57)	0.062
Respiratory diseases, n (%)	17 (22.37)	11 (64.70)	6 (35.29)	0.021
Nephropathy, n (%)	9 (11.84)	8 (88.89)	1 (11.11)	>0.9
Infection within six months of full vaccination, n (%)	18 (33.33)	14 (77.78)	4 (22.22)	0.5
Vaccination				0.8
0, n (%)	13 (17.11)	10 (76.92)	3 (23.08)	
1 dose, n (%)	0 (0)	0 (0)	0 (0)	
2 doses, n (%)	9 (11.84)	8 (88.89)	1 (11.11)	
3 doses, n (%)	41 (53.95)	34(82.92)	7 (17.07)	
4 doses, n (%)	13 (17.11)	12 (92.30)	1 (8.33)	
tixagevimab/cilgavimab prophylaxis, n (%)	11 (14.47)	10 (90.9)	1 (9.1))	>0.9
infection within six months of prophylaxis with tixagevimab/cilgavimab, n (%)	7 (9.21)	6 (85.71)	1 (14.28)	>0.9
Early therapy, n (%)	19 (25)	18(94.73)	1(5.26)	0.3
CRP (mg/dL with normal values <0.5 mg/dL)				0.6
Mean (SD)	10.16 (8.12)	9.95 (7.92)	11.31 (9.38)	
Median [25%-75%]	8.03 [4.38-13.78]	7.95 [4.38-13.48]	8.51 [5.69-14.02]	
Hematologic therapy				0.4
Chemotherapy alone, n (%)	19 (25)	15 (78.95)	4 (21.05)	
Ibrutinib, n (%)	8 (10.52)	5 (62.50)	3 (37.50)	
Including anti-CD20, n (%)	18 (23.68)	17 (94.44)	1 (5.56)	
Stem cell transplantation, n (%)	5 (6.57)	4 (80)	1 (20)	
Including anti-Bcl-2, n (%)	3 (3.94)	3 (100)	0 (0)	
IMiDs +/- protease inhibitor, n (%)	6 (7.89)	6 (100)	0 (0)	
CAR-T, n (%)	0 (0)	–	–	
No therapy, n (%)	17 (22.36)	14 (82.4)	3 (17.6)	
Infection within 1 year of hematologic therapy, n (%)	52 (68.4)	43(79.7)	9 (17.3)	>0.9

MoAb, monoclonal antibodies; Triple, triple therapy (two antivirals + one MoAb); HL, Hodgkin lymphoma; NHL, non-Hodgkin lymphoma; CML, chronic myelogenous leukemia; CLL, chronic lymphocytic leukemia; AML, acute myelogenous leukemia; MM, multiple myeloma; MF, myelofibrosis; MDSs, myelodysplastic syndrome; CRP, C-reactive protein; IMiDs, Thalidomide immunomodulatory analogs; CAR-T, Chimeric Antigen Receptor T-cell therapy. Bold values in the tables indicate statistically significant values.

**Figure 2:**
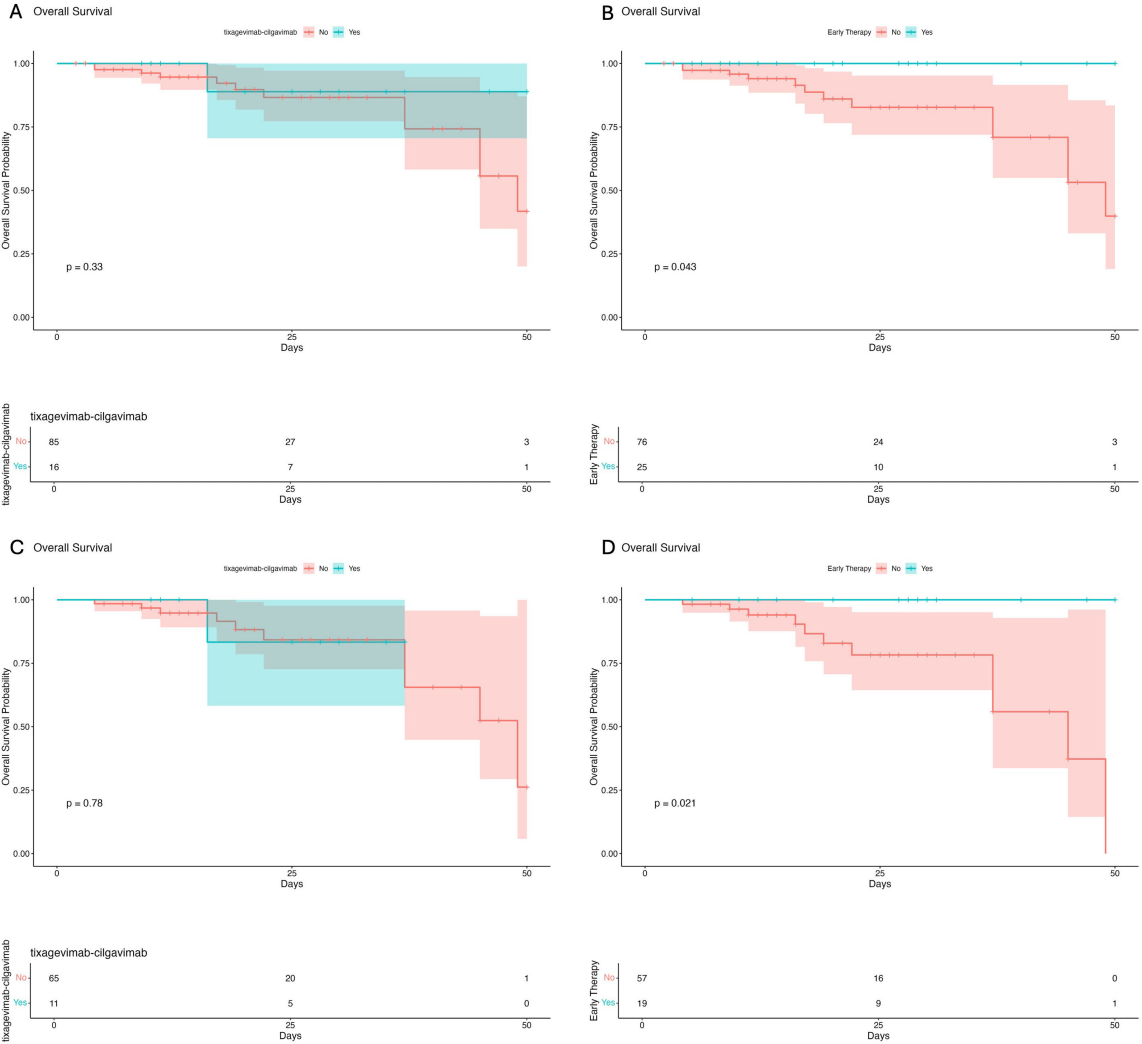
Survival analysis in the overall population of patients receiving tixagevimab-cilgavimab **(A)** and early therapy **(B)** before hospital admission. Survival analysis in the pneumonia subgroup of patients treated with tixagevimab-cilgavimab **(C)** and early therapy **(D)** before hospital admission.

Survival analysis using Kaplan-Meier curves was performed to better study the impact of preventive interventions on in-hospital survival in the overall population (**Figures 2A, B**) and in pneumonia patients (**Figures 2C, D**). We observed significant higher survival rates in patients treated with early therapy in the overall population (log-rank p= 0.043) and in the pneumonia subgroup (log-rank p= 0.021).

The trend of Kaplan-Meier curves also suggests higher survival rates in patients treated with tixagevimab-cilgavimab prophylaxis, although the limited sample size prevents the observation of statistical significance (**Figures 2A, C**).

Furthermore, no statistically significant difference was observed regarding the time to viral clearance in patients treated with early therapy (p=0.514) or, pre-exposure prophylaxis with tixagavimab-cilgavimab (p=0.9338).

To assess the impact of different anti-SARS-CoV-2 in-hospital therapies on mortality and time to viral clearance, pneumonia patients who underwent therapy (n=71) for SARS-CoV-2 were divided into four groups based on the administered treatment:

- 3 patients were treated with MoAb in monotherapy.
- 27 patients were treated with remdesivir (either for five days or for 10 days);
- 34 patients received therapy a combination of MoAbs and remdesivir;
- 7 patients received triple therapy with dual AV therapy, intravenous remdesivir (for 10 days), and oral AV with nirmatrelvir-ritonavir (in four cases extended for 10 days) and MoAb.

None of the patients treated with triple therapy died; in contrast, 18.51% of the population treated with remdesivir and 20.59% of those who received remdesivir and MoAb died. In the MoAb group, no deaths were recorded; however, this data may be influenced by the extremely limited sample size of the group. The characteristics of patients who underwent triple therapy were reported in **Table 3**. To evaluate whether hematologic comorbidities or other clinical characteristics could serve as confounding factors for mortality in patients treated for COVID-19 pneumonia, we compared overall comorbidities, general characteristics, oncohematologic conditions, and treatments across different therapy groups. Specifically, we analyzed remdesivir versus triple therapy and remdesivir plus a monoclonal antibody versus triple therapy in **Tables 4**, **5**. The comparison of treatment groups revealed no significant differences in overall comorbidities, general characteristics, oncohematologic conditions, or specific oncohematologic treatments. However, a significant age difference was observed, with patients receiving triple therapy being younger than those in the remdesivir and remdesivir plus monoclonal antibody groups. Nonetheless, the mean age remained above 65 years across all treatment groups. Regarding the second endpoint we analyzed the viral persistence in our population considering viral clearance from the beginning of first positive swab as well as viral clearance following the administration of the SARS-CoV-2 therapy, as done in other studies (26, 31).

**Table 3:** Clinical characteristics of subjects treated with triple therapy.

Sex	Age (years)	Hematological malignancy	Comorbidities	Ongoing Hematological therapy	Vaccination (doses)	Prophylaxis with Tixagevimab/Cilgavimab	Early therapy	SARS-CoV-2 related Pneumonia	Hospitalization time (days)	Negativization time (overall, days)	Negativization time (after triple therapy, days)
M	63	NHL	Yes (respiratory disease)	Yes	3	No	No	Mild	21	22	9
M	60	NHL	No	No	3	No	No	Mild	14	22	3
F	63	NHL	No	Yes	3	Yes	No	Moderate	37	10	6
M	69	NHL	Yes (cardio-vascular diseases, age >65 yo)	Yes	3	Yes	No	Mild	25	8	7
F	67	NHL	Yes (cardio-vascular diseases, diabetes, age >65 yo)	Yes	4	Yes	Yes, nirmatrelvir/ritonavir	Mild	28	23	4
M	72	NHL	Yes (cardio-vascular diseases, diabetes, age >65 yo)	Yes	4	No	Yes, sotrovimab	Mild	12	11	9
M	64	CLL	No	Yes	4	No	No	Mild	16	15	4

M, male; F, female; NHL, non-Hodgkin lymphoma; CLL, chronic lymphocytic leukemia; yo, years old.

**Table 4:** Comparison of remdesivir + MoAb and triple therapy groups.

Characteristic	All patients n (%) = 41 (100)	Remdesivir +MoAb n (%) = 34 (82.9)	Triple therapy n (%) = 7 (17.1)	P-value
Sex, female, n (%)	14.00 (34.15)	12.00 (85.7)	2.00 (14.3)	>0.9
Age at hospitalization (years)				**0.02**
Mean (SD)	71.59 (9.71)	72.85 (10.08)	65.43 (4.12)	
Median [25%-75%]	72.00 [65.00-80.00]	74.00 [67.00-82.00]	64.00 [63.00-69.00]	
Length of hospital stay (days)				0.4
Mean (SD)	20.34 (11.37)	20.03 (11.92)	21.86 (8.86)	
Median [25%-75%]	16.00 [11.00-28.00]	15.00 [10.00-29.00]	21.00 [14.00-28.00]	
Time to viral clearance (days)				0.1
Mean (SD)	27.17 (22.06)	30.00 (23.71)	15.86 (6.41)	
Median [25%-75%]	22.00 [15.00-31.00]	24.50 [15.50-32.50]	15.00 [10.00-22.00]	
Difference between the start of therapy and first negative swab (days)				0.07
Mean (SD)	12.73 (10.71)	14.12 (11.25)	6.00 (2.45)	
Median [25%-75%]	9.00 [5.00-16.00]	11.00 [5.00-22.00]	6.00 [4.00-9.00]	
Hematological malignancy				
HL, n (%)	1.00 (2.44)	1.00 (100)	0.00 (0.00)	>0.9
NHL, n (%)	23.00 (56.10)	17.00 (73.9)	6.00 (26.1)	0.11
AML, n (%)	2.00 (7.32)	2.00 (100)	0.00 (0.00)	>0.9
CML, n (%)	1.00 (2.44)	1.00 (100)	0.00 (0.00)	>0.9
CLL, n (%)	9.00 (21.95)	8.00 (88.9)	1.00 (11.1)	>0.9
MM, n (%)	3.00 (7.32)	3.00 (100)	0.00 (0.00)	>0.9
MF, n (%)	0			
MDSs, n (%)	2.00 (4.88)	2.00 (100)	0.00 (0.00)	>0.9
Comorbidity				
Age>65 years old, n (%)	31.00 (75.61)	28.00 (90.3)	3.00 (9.7)	**0.047**
Cardiovascular disease, n (%)	22.00 (53.66)	19.00 (86.3)	3.00 (13.7)	0.7
Diabetes, n (%)	10.00 (24.39)	8.00 (803)	2.00 (20)	>0.9
Respiratory diseases, n (%)	8.00 (19.51)	7.00 (87.5)	1.00 (12.5)	>0.9
Nephropathy, n (%)	6.00 (14.63)	6.00 (100)	0.00 (0.00)	0.6
Vaccination				0.6
0, n (%)	5.00 (12.20)	5.00 (14.71)	0.00 (0.00)	
1 dose, n (%)	0			
2 doses, n (%)	3.00 (7.32)	3.00 (8.82)	0.00 (0.00)	
3 doses, n (%)	23.00 (56.10)	19.00 (55.88)	4.00 (57.14)	
4 doses, n (%)	10.00 (24.39)	7.00 (20.59)	3.00 (42.86)	
tixagevimab/cilgavimab prophylaxis, n (%)	10.00 (24.39)	7.00 (70)	3.00 (30)	0.3
Early therapy, n (%)	9.00 (21.95)	7.00 (77.8)	2.00 (22.2)	0.6
Hematologic therapy				0.7
Chemotherapy alone, n (%)	11.00 (27.50)	9.00 (81.8)	2.00 (18.2)	
Ibrutinib, n (%)	4.00 (10.00)	3.00 (75)	1.00 (25)	
Including anti-CD20, n (%)	10 (24.4)	6.00 (60)	3.00 (40)	
Stem cell transplantation, n (%)	4.00 (10.00)	3.00 (75)	1.00 (25)	
Including anti-Bcl-2, n (%)	3.00 (7.50)	3.00 (100)	0.00 (0.00)	
IMiDs +/- protease inhibitor, n (%)	1.00 (2.50)	1.00 (100)	0.00 (0.00)	
CAR-T, n (%)	0			
No therapy, n (%)	8 (17.50)	8.00 (100)	0.00 (0.00)	
Infection within one year of hematologic infection, n (%)	28.00 (84.85)	22.00 (84.62)	6.00 (85.71)	>0.9
Outcome, n (%)				0.9
Death, n (%)	7.00 (17.07)	7.00 (20.59)	0.00 (0.00)	

MoAb, monoclonal antibodies; Triple, triple therapy (two antivirals + one MoAb); HL, Hodgkin lymphoma; NHL, non-Hodgkin lymphoma; CML, chronic myelogenous leukemia; CLL, chronic lymphocytic leukemia; AML, acute myelogenous leukemia; MM, multiple myeloma; MF, myelofibrosis; MDSs, myelodysplastic syndrome; IMiDs, Thalidomide immunomodulatory analogs; CAR-T, Chimeric Antigen Receptor T-cell therapy. Bold values in the tables indicate statistically significant values.

**Table 5:** Comparison of remdesivir and triple therapy group.

Characteristic	All patients n (%) = 34 (100)	Remdesivir n (%) =27 (79,4)	Triple therapy n (%) =7 (20.6)	P-value
Sex, female, n (%)	16.00 (47.06)	14.00 (87.5)	2.00 (12.5)	0.4
Age at hospitalization (years)				**0.009**
Mean (SD)	71.65 (8.67)	73.26 (8.85)	65.43 (4.12)	
Median [25%-75%]	72.00 [67.00-78.00]	74.00 [69.00-80.00]	64.00 [63.00-69.00]	
Length of hospital stay (days)				0.6
Mean (SD)	21.68 (13.06)	21.63 (14.08)	21.86 (8.86)	
Median [25%-75%]	17.50 [12.00-31.00]	15.00 [11.00-33.00]	21.00 [14.00-28.00]	
Time to viral clearance (days)				0.15
Mean (SD)	31.48 (25.57)	36.45 (27.44)	15.86 (6.41)	
Median [25%-75%]	22.00 [12.00-44.00]	28.00 [13.00-65.00]	15.00 [10.00-22.00]	
Difference between the start of therapy and first negative swab (days)				**0.005**
Mean (SD)	21.21 (21.07)	25.15 (22.00)	6.00 (2.45)	
Median [25%-75%]	12.00 [6.00-34.00]	17.00 [8.00-37.00]	6.00 [4.00-9.00]	
Hematological malignancy				
HL, n (%)	0			
NHL, n (%)	15.00 (44.12)	9.00 (60)	6.00 (40)	**0.028**
AML, n (%)	2.00 (5.88)	2.00 (100)	0.00 (0)	>0.9
CML, n (%)	1.00 (2.94)	1.00 (100)	0.00 (0)	>0.9
CLL, n (%)	10.00 (29.41)	9.00 (90)	1.00 (10)	0.6
MM, n (%)	5.00 (14.71)	5.00 (100)	0.00 (0)	0.6
MF, n (%)	1.00 (2.94)	1.00 (100)	0.00 (0)	>0.9
MDSs, n (%)	0			
Comorbidity				
Age>65 years old, n (%)	27.00 (79.41)	24.00 (88.9)	3.00 (11.1)	**0.020**
Cardiovascular disease, n (%)	23.00 (67.65)	20.00 (87)	3.00 (13)	0.2
Diabetes, n (%)	10.00 (29.41)	8.00 (80)	2.00 (20)	>0.9
Respiratory diseases, n (%)	7.00 (20.59)	6.00 (85.7)	1.00 (14.3)	>0.9
Nephropathy, n (%)	0			
Vaccination				0.2
0, n (%)	5.00 (14.71)	5.00 (100)	0.00 (0.00)	
1 dose, n (%)	0			
2 doses, n (%)	3.00 (8.82)	3.00 (100)	0.00 (0.00)	
3 doses, n (%)	20.00 (58.82)	16.00 (80)	4.00 (20)	
4 doses, n (%)	6.00 (17.65)	3.00 (50)	3.00 (50)	
tixagevimab/cilgavimab prophylaxis, n (%)	4.00 (11.76)	1.00 (25)	3.00 (75)	**0.021**
Early therapy, n (%)	10.00 (29.41)	8.00 (80)	2.00 (20)	>0.9
Hematologic therapy, n (%)				0.2
Chemotherapy alone, n (%)	7.00 (20.59)	5.00 (71.4)	2.00 (28.6)	
Ibrutinib, n (%)	5.00 (14.71)	4.00 (80)	1.00 (20)	
Including anti-CD20, n (%)	9.00 (26.47)	6.00 (66.7)	3.00 (33.3)	
Stem cell transplantation, n (%)	1.00 (2.94)	0.00 (0)	1.00 (100)	
Including anti-Bcl-2, n (%)	4.00 (11.76)	4.00 (10)	0.00 (0)	
IMiDs +/- protease inhibitor, n (%)	0			
CAR-T, n (%)	0			
No therapy, n (%)	8.00 (23.53)	8.00 (100)	0.00 (0)	
Infection within one year of hematologic infection, n (%)	25.00 (92.59)	19.00 (76)	6.00 (24)	0.5
Outcome, n (%)				0.6
Death, n (%)	5.00 (14.71)	5.00 (100)	0.00 (0)	

MoAb, monoclonal antibodies; Triple, triple therapy (two antivirals + one MoAb); HL, Hodgkin lymphoma; NHL, non-Hodgkin lymphoma; CML, chronic myelogenous leukemia; CLL, chronic lymphocytic leukemia; AML, acute myelogenous leukemia; MM, multiple myeloma; MF, myelofibrosis; MDSs, myelodysplastic syndrome; IMiDs, Thalidomide immunomodulatory analogs; CAR-T, Chimeric Antigen Receptor T-cell therapy. Bold values in the tables indicate statistically significant values.

**Figure 3:**
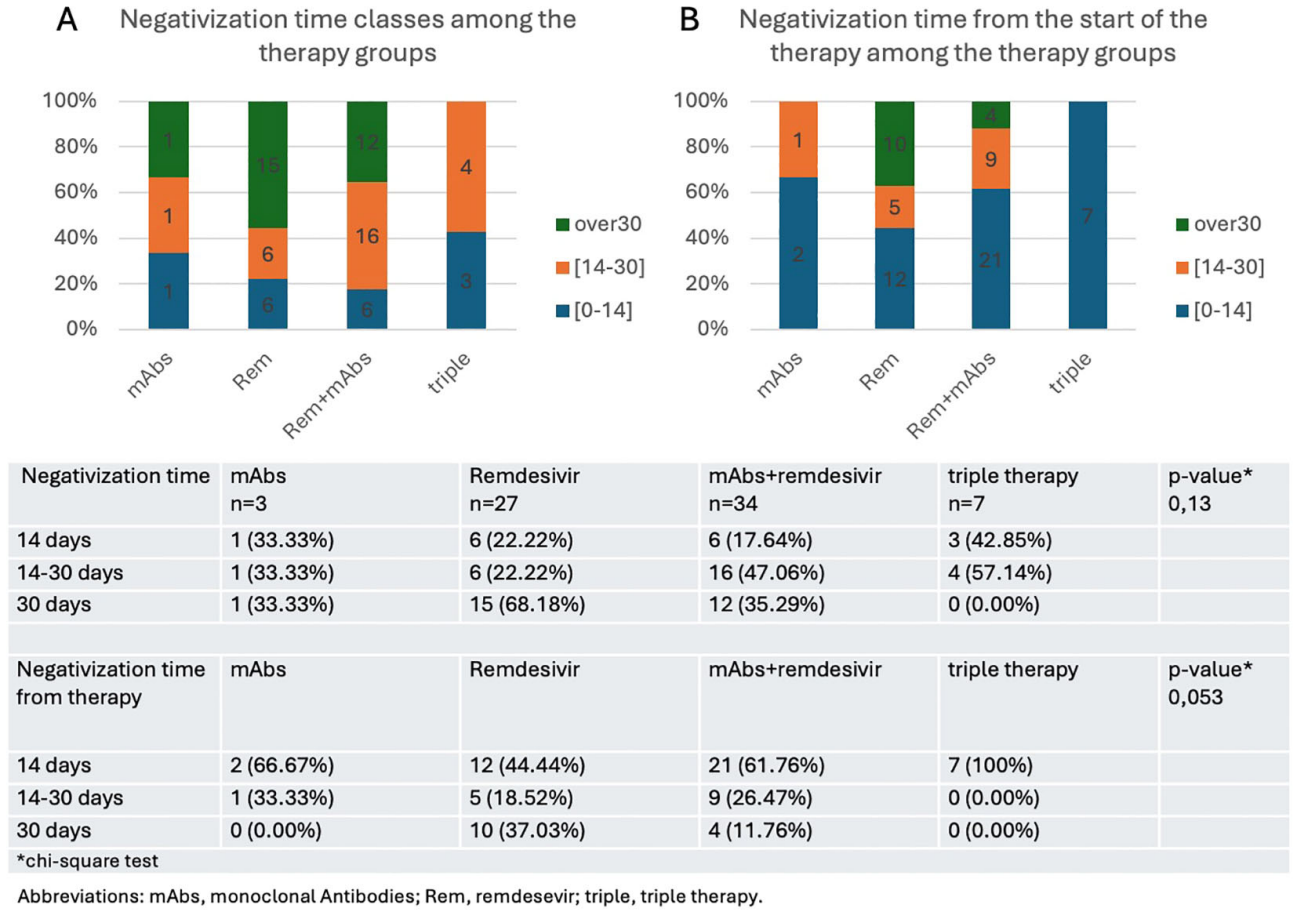
Time to negativization from the onset of infection **(A)** or from the start of therapy **(B)** expressed as rate of patients who achieve a negative swab in less than 14 days, between 14 and 30 days, or in more than 30 days.

From these data sets, it is evident that, considering the global negativization time, 42.8% of patients treated with triple therapy fall into the ≤14 days category, while 57.14% fall into the ≤30 days category and none of the patients who received triple therapy had a negativitation time longer than 30 days. Meanwhile, when considering the negativization time interval from the initiation of therapy, 100% of subjects treated with triple therapy experienced negativization within 14 days. These differences confirm the significant role of triple therapy in improving viral clearance compared to other treatment groups (**Figures 3A, B**). The trend of viral clearance in different therapy groups suggests that triple therapy is the most effective option, followed by combination therapy with MoAb and remdesivir, and subsequently by monotherapy with remdesivir. The chi-square test, which was performed to analyze the relationship between the four different therapy groups and the overall negativization time—categorized into three distinct groups (≤14 days, between 14 and 30 days, and over 30 days)—did not reveal a statistically significant association, as indicated by a p-value of 0.13. Conversely, when the chi-square test was applied to assess the association between the time to negativization measured specifically from the initiation of therapy within the four therapy groups the p-value was 0.053.

An initial Mann-Whitney test revealed that patients treated with triple therapy showed a significant reduction in the time to viral clearance from the first day of the evaluated therapy (6 days [IQR 4;9]), compared to patients treated with only intravenous AVs (17 days [IQR 8;37]) (p=0.03). The results of the Kruskal-Wallis test on the statistical significance of differences in the clearance times of different therapies showed a significant difference (p=0.016). To control for the family-wise error rate when performing multiple comparisons, we used a paired Wilcoxon test, with Bonferroni adjustment, which identified that the significant difference was specifically between the remdesivir and triple therapy group (p=0.016 in that case). In our case, in addition to the previously mentioned median virological clearance times for both the remdesivir-only therapy and the triple therapy, the combined treatment of remdesivir and monoclonals reported a median time of 11 days with an interquartile range (IQR) of 5 to 22.75 days.

**Figure 4:**
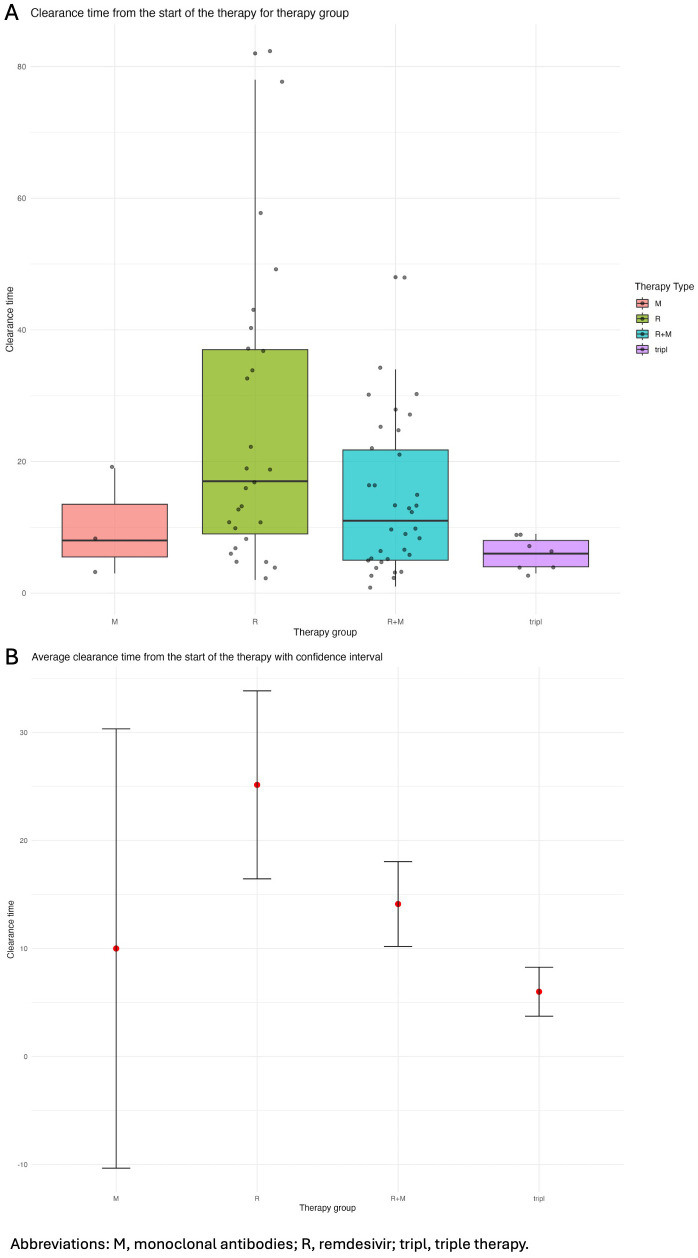
**(A)** Boxplot representing the distribution of clearance time following the administration of the SARS-COV-2 therapy; **(B)** Average clearance time from the start of therapy with confidence interval.

The medians suggest a decreasing trend in clearance time that correlates with an increase in the number of treatments used: from one to triple therapy. However, the relatively short median clearance time for the monoclonal therapy alone (8 days [IQR 3;19]) appears to contradict this. It must be noted that this median was calculated from a small number of cases, where individual differences may have skewed the data. The confidence intervals shown in **Table 6**, **Figure 4** confirm our data on the time to viral clearance. Thus, while the paired test did not reach statistical significance for the remdesivir + MoAb group, the trend in medians suggests a potential practical significance (**Table 6**, **Figure 4**).

**Table 6:** impact on viral clearance of different SARS-CoV-2 therapies in the pneumonia population.

Subpopulation n (%) = 71 (100)			Remdesivir+MoAb n (%) = 34 (47.9)	Triple n (%) = 7 (9.9)
	MoAb n (%) = 3 (4.2)	Remdesivir n (%) = 27 (38)		
Clearance time (days)				
Mean (SD) [CI]	19.00 (11.53) [-9.65-47.6]	36.45 (27.44) [24.3-48.6]	30.00 (23.71) [20.8-39.2]	15.86 (6.41) [9.92-21.8]
Median [Q1-Q3]	20.00 [7.00-30.00]	28.00 [13.00-65.00]	24.50 [15.50-32.50]	15.00 [10.00-22.00]
Clearance time starting from the beginning of the therapy (days)				
Mean (SD) [CI]	10.00 (8.19) [-10.3-30.3]	25.15 (22.00) [16.4-33.8]	14.12 (11.25) [10.2-18.0]	6.00 (2.4 5) [3.73-8.27]
Median [Q1-Q3]	8.00 [3.00-19.00]	17.00 [8.00-37.00]	11.00 [5.00-22.00]	6.00 [4.00-9.00]

MoAbs, monoclonal antibodies; Triple, triple therapy (two antivirals + one MoAb).

However, to better evaluate whether the drugs used may have influenced treatment efficacy over the three years of the study, we assessed whether the outcome varied in the population with pneumonia treated with monoclonal antibodies, including monotherapy with MoAb, association therapy with MoAb and remdesivir and triple therapy, depending on the timing of treatment initiation. The therapy start date was used as it appeared to be a variable directly associated with the treatment administered. For this reason, therapy start dates were grouped by quarter, and a Fisher’s exact test was performed on the outcome, which was not significant (p = 0.383). The same test was applied using monthly grouping (p= 0.696) and semi-annual grouping (p = 0.496).

To rule out the possibility that different viral variants influenced the outcome, we analyzed the outcome based on the date of the first positive swab in patients with pneumonia. These dates were grouped quarterly and a Fisher’s exact test was performed, yielding non-significant results (p = 0.769). Similar analyses using monthly (p = 0.750) and semi-annual (p = 0.809) groupings also showed no significant associations.

In our study, the number of vaccinations undergone did not significantly impact the time to negativitazion in the vaccinated population. The overall population was stratified in three groups: patients who did not receive vaccination, patients with one or two doses of vaccine and patients who received booster dosages (3 or more doses). Concerning the booster dosages (68 patients): 15 patients (22.05%) cleared the virus within the 14 days and 30 patients (44.11%) cleared the virus between 14 and 30 days. Interestingly only 23 patients (33.82%) took more than 30 days to clear the virus compared to 43.75% unvaccinated patients and 52.94% of those with one or two doses of vaccines (**Table 7**).

**Table 7:** impact of vaccination doses on viral clearance on overall population.

Negativization time Overall population n (%) =101 (100)	<14 days	14-30 days	>30 days
0 dose n (%) =16 (15.8)	3 (18.75)	6 (37.5)	7 (43.75)
1-2 doses n (%) =17 (16.8)	5 (29.41)	3 (17.65)	9 (52.94)
3 or more doses n (%)= 68 (67.3)	15 (22.06)	30 (44.12)	23 (33.82)

Regarding the safety of COVID-19 treatments, 23% of patients (n=3/13) treated with nirmatrelvir/ritonavir experienced reversible dysgeusia, while 7% (n=1/13) developed diarrhea without subsequent dehydration or electrolyte imbalances. A mild and asymptomatic increase in transaminase levels was observed in 3% (n=3/91) of patients treated with remdesivir, but it was not deemed significant enough to warrant discontinuation of treatment. None of the observed adverse effects were severe. No allergic reactions were observed in hematologic patients treated with antivirals or monoclonal antibodies. Additionally, to evaluate the overall incidence of adverse effects associated with different treatment options, we analyzed the total number of drug administrations across distinct anti-SARS-CoV-2 therapeutic strategies (prophylaxis, early treatment, and treatment in hospitalized patients with or without pneumonia), as reported in **Supplementary Table 1**.

## 4 Discussion

Clinical experience and scientific literature support the notion that oncohematological patients with COVID-19 pose a challenge for clinicians due to their increased risk of progressing to severe forms of the disease and experiencing worse outcomes (32).

Studies have consistently revealed that individuals with hematological malignancies are at a significantly elevated risk for hospitalization, intensive care unit admission, mechanical ventilation, and increased mortality rates. These outcomes are also higher compared to patients with solid tumors (33).

On one hand, our research does not demonstrate a significant positive impact on mortality across the four different therapeutic approaches used to treat patients with COVID-19 pneumonia.

On the other hand, our results provide evidence that hospitalized patients with COVID-19 pneumonia, who were previously treated with early therapy targeting SARS-CoV-2 (32.07%) have a higher survival rate (p=0.021) compared to hospitalized patients who did not receive early therapy before admission. The positive effect on the survival rate in oncohematological patients treated with early therapy was previously confirmed with regard to the use of MoAb, with a mortality of 3.3% due to COVID-19, according to some sources in literature (17). Subsequently, early therapy can play a significant role in changing the natural history of SARS-CoV-2 infection in immunocompromised patients, in which vaccination is not always sufficient (34). However, according to other authors, early therapy with MoAb has been identified as an independent factor of therapeutic failure and prolonged infection in this fragile patients’ category. In addition, early therapy in such patients was associated with significantly higher rates of progression towards severe forms of COVID-19 (26%), with a COVID-19 mortality rate of 3.4% (10). In our study the choice of specific early therapy was made by infectious disease specialists, taking into account patients’ comorbidities, potential drug interactions and logistical considerations. In particular, nirmatrelvir/ritonavir is contraindicated in patients with < 30 ml/min renal clearance and in patients with severe hepatic impairment (Child-Pugh C). Moreover, ritonavir is a strong inhibitor of Cytochrome CYP3A, making its use contraindicated with drugs metabolized by CYP3A, such as statins and antiarrhythmics. Conversely, a short 3-day course of remdesivir is not contraindicated in patients with renal insufficiency or those undergoing hemodialysis. However, it is not recommended in patients with severe hepatic insufficiency (Child-Pugh C), and its daily intravenous administration presents logistical challenges (35). MoAbs have fewer limitations.

Moreover, remdesivir is a nucleotide analogue who targets RNA-dependent RNA polymerase (RdRp) enzyme in order to inhibit viral replication (36). Nirmatrelvir inhibits the SARS-CoV-2 3CL protease, an enzyme essential for viral replication, while coadministration with ritonavir slows its metabolism, prolonging its activity in the body and maintaining higher concentrations (37). Moreover, MoAbs prevent SARS-CoV-2 from entering human cells by targeting various epitopes on the spike protein, such as the receptor-binding domain (RBD) or the S2 subunit, thereby blocking its interaction with the angiotensin-converting enzyme 2 (ACE2) on the cell surface (38).

Clinical manifestations of COVID-19 are mainly driven by the biphasic nature of the disease, which begins with a phase of viral replication and toxicity, followed by a second phase characterized by an inflammatory response. The latter is responsible for the progression of the disease and its severity. For this reason, early therapy is administered in the first phase of COVID-19 to prevent potential complications such as respiratory failure and death (39). The effectiveness of early therapy in reducing disease progression and mortality is described in several studies: a large retrospective study showed that early therapy reduces disease progression in a real-life scenario (40). Another study demonstrated that nirmatrelvir-ritonavir reduced hospitalization rate and mortality among patients 65 years and older (41). Conversely Najjar-Debbiny et al. demonstrated that nirmatrelvir-ritonavir reduced disease progression and mortality in overall population in omicron wave (42) Another study showed that early remdesivir prevented disease progression and death among outpatients (43).

In a Phase-III controlled randomized trials on molnupiravir and nirmatrelvir/ritonavir, oncohematological patients are poorly represented compared to the general population and patients with metabolic disorders (44). For this reason, studies with a larger number of oncohematological patients treated with early therapy and, or oral AV are needed to assess their effectiveness.

Regarding COVID-19 prophylaxis, in our study tixagevimab/cilgavimab was administered to a limited number of patients. Specifically, only 16 patients received tixagevimab/cilgavimab and all of them were hospitalized at the time of the drug’s administration. This is noteworthy because COVID-19 prophylaxis with tixagevimab/cilgavimab is typically given exclusively to outpatients. In any case, it appeared to have no effect on mortality, patient susceptibility to infection, or viral clearance. In contrast, other studies have proven the effectiveness of tixagevimab/cilgavimab in reducing the severity of SARS-CoV-2 infection and mortality in fragile patients, suggesting its efficacy for immunocompromised individuals (45, 46). In one study, patients with multiple myeloma who received prophylaxis with tixagevimab/cilgavimab showed a high neutralizing antibody titer, with a low incidence of SARS-CoV-2 infection and hospitalization during the follow-up (47).

Perhaps, in order to maximize the protective effect of tixagevimab/cilgavimab, a combination of preventive strategies (e.g., vaccinations, pre-exposure prophylaxis and early therapies) should be considered (48).

Existing therapies, when used singularly, often seem to be insufficient in treating COVID-19 and clearing SARS-CoV-2 (14, 15, 27). In our study, patients treated with triple therapy had a lower average negativization time than other treatment options. Additionally, there was a significant reduction in time to negativization (6 days [IQR 4;9]) in patients treated with triple therapy compared to patients treated with intravenous AV alone (17 days [IQR 8;37) (p=0.003). This demonstrates the ability of triple therapy in more effectively achieving viral clearance. Thus, our research highlights that adopting combined therapeutic approaches against severe COVID-19 in oncohematological patients can improve outcomes related to SARS-CoV-2 infection.

Existing literature on the effectiveness of therapeutic strategies for SARS-CoV-2 infections in oncohematological patients is limited, particularly regarding combination therapies. The few published studied consist mostly in case reports (49, 50) and case series (21–25, 51). In one study, 10 oncohematological patients with COVID-19 pneumonia and prolonged infection were treated with a combination of AV and MoAb therapy. Of these, 5 received two different consecutive AVs: in all cases remdesivir therapy was changed to molnupiravir or nirmatrelvir/ritonavir. None of these patients developed relapse of infection (21). Another study indicates that combination of AV and MoAb therapy in oncohematological patients is safe and can effective (22). In another study combination therapy of remdesivir and molnupinavir was administered to 5 patients with severe COVID-19; in all cases there was a complete clinical improvement (23). Another study demonstrated that 11 out of 14 immunocompromised patients treated with triple therapy with remdesivir and nirmatrelvir/ritonavir or molnupinavir plus tixagevimab/cilgavimab developed virological and clinical resolution (24). These results align with the findings of Gentile I et al. demonstrating the effectiveness of triple therapy in achieving virological and clinical resolution in early-stage COVID-19 patients with oncohematological conditions (29).

Other studies focused on viral clearance in patients with prolonged COVID-19, or ‘persistant COVID-19’. For Meijer et al. patients with persistent COVID-19 were those displaying signs and symptoms of COVID-19 (fever ≥38.0 °C or new respiratory symptoms), for at least 14 days after the initial diagnosis. In their study population 73% of patients treated with a 5-day course of remdesivir and nirmatrelvir/ritonavir, with or without the addition of tixagevimab/cilgavimab, effectively cleared the virus by the end of treatment and the rest had an increase in PCR cycle threshold (CT) values (51). In another study, the same drug combination was given for 10 days, yielding similar results in terms of viral clearance, as all patients showed viral clearance after 9 days (25).

Dioverti et al. demonstrated promising results in three hematologic patients with prolonged SARS-CoV-2 infection and B-cell depletion treated with AV therapy. Specifically, remdesivir was given for 5 days along with 1200/1200 mg casirivimab/imdevimab (49). The theoretical rationale behind this was to target the virus via different mechanisms simultaneously. Indeed, remdesivir has been proven to reduce viral replication, while casirivimab/imdevimab supports the immune response through neutralizing antibodies against SARS-CoV-2 in patients unable to produce their own neutralizing antibodies due to B cell depletion. Moreover, in immunocompromised patients with B cell depletion, combination multi-target therapy (AV or dual AV plus antibody-based therapies) has been shown to be more effective than monotherapy in preventing persistent viral shedding and/or severe SARS-CoV-2 infection (31).A case report describes the use of dual AV therapy in a patient with chronic lymphocytic leukemia, who had previously received early therapy with nirmatrelvir/ritonavir and remdesivir for 10 days. Given the subsequent prolonged infection and persistence of bilateral interstitial pneumonia, a simultaneous 10-day administration of nirmatrelvir/ritonavir and remdesivir was initiated, leading to clinical resolution in 9 days and rapid viral clearance without relapses at ten months of follow-up (50).

Our data are in agreement with those of a recent case study conducted on 22 immunocompromised and oncohematological patients with prolonged SARS-CoV-2 infection, who were treated with combination therapy. The majority (81%) received triple therapy (a 10 day-course of remdesivir, in association with oral AV (nirmatrelvir/ritonavir or molnupiravir in case of renal failure) and MoAb (directed against the dominant circulating variant), while the remaining patients were treated with dual AV therapy. The results show that both early virological response (within 14 days of treatment) and late virological clearance were higher in the combination therapy group. In this study, combination therapy proved effective in reducing the time to viral clearance and clinical response compared to AVs alone (26). Finally, a case series published in 2022 describes two patients with COVID-19 pneumonia and concomitant B cell depletion due to Rituximab therapy who had been successfully treated with triple therapy consisting in sotrovimab, remdesivir and nirmatrelvir/ritonavir (52).

Clearly, combining AV agents with different mechanisms of action and MoAb targeting the spike protein may provide significant benefits, especially in patients lacking humoral immunity, where AV therapy alone may be insufficient to resolve the infection. In both the aforementioned study and ours, triple therapy was administered to patients with prolonged SARS-CoV-2 infection. In our study the triple therapy was used as rescue therapy after the failure of therapy with a 10-day course of remdesivir, with or without MoAb.

Clearly, our study highlights patients whose SARS-CoV-2 treatment was tailored to the individual on the basis of symptoms and persisting positivity according to antigenic swabs. Indeed, in some cases of prolonged infection, the standard duration of oral AV therapy was doubled and/orintravenous therapy with remdesivir was prolonged with respect to the intended treatment plan (up to a total of 30 days). In these patients, outcomes were generally favorable. This suggests that personalized and complex treatment strategies are needed for this category of vulnerable patients with prolonged infections.

Moreover, in our study population, advanced age (>65 years) was an unfavorable prognostic factor (p=0.008). The EPICOVIDEHA cohort collected data on 3801 oncohematological patients with SARS-CoV-2 infection: analysis showed that factors such as age, active hematological malignancies, cardiovascular diseases, renal and hepatic pathologies, smoking and admission to ICU, were related to high mortality (18).

Advanced age has been shown to be associated with increase mortality in many other studies involving oncohematological patients with COVID-19 (53). Advanced age is also considered a risk factor for progression to severe disease even in patients not affected by oncohematological malignancies. Our data show that chronic respiratory diseases, such as asthma and chronic obstructive pulmonary disease, are associated with a worse prognosis.

Chronic respiratory diseases are a significant risk factor in the progression to severe forms of disease in patients with SARS-CoV-2 infection. In fact, together with age, they constitute one of the criteria considered for the beginning of early therapy in patients with SARS-CoV-2. A study carried out on a large sample of patients with hematological neoplasms shows that smoking is associated with higher mortality (54).

This study had several limitations. First, its retrospective design may have introduced selection and information biases. Additionally, the relatively small sample size limits the statistical power of our findings. A further limitation was the heterogeneity in both the study sample and the treatment options administered during the study period and their effect on different viral variants. The latter is attributable to the data being collected from two different hospitals over a three-year span. The extended duration of the study allowed for the inclusion of different phases of the COVID-19 epidemic, characterized by varying dominant viral variants. Until June 2021, data from the Italian National Institute of Health indicated that the alpha variant was predominant in Italy. From July 2021 to December 2022, the delta variant prevailed. The first case of the omicron variant was identified in Italy on November 27, 2021, and from January 3, 2022, it has remained the dominant strain (55).

It is well established that different variants exhibit varying susceptibility to therapeutic agents, particularly MoAbs. For example, MoAbs like casirivimab and imdevimab (approved in Europe in 2021) were effective against the alpha and delta variants but showed significantly reduced efficacy against the omicron variant due to mutations in the spike protein. Similarly, sotrovimab, initially used against omicron, experienced decreased efficacy as further mutations emerged in later sublineages. In fact, our results for the MoAb group may have been influenced by the small sample size and the timing of treatment, as two of the three patients in this group were treated during the period when the Delta variant was dominant, a strain against which monoclonal antibodies demonstrated greater efficacy (56).

The heterogeneity of the study was further compounded by the evolving availability of treatment options over the study period. MoAbs and AVs became available at different times. MoAbs targeting the spike protein of SARS-CoV-2 became available in May 2021. However, their usage was inconsistent throughout the study period. Oral antiviral therapies (nirmatrelvir-ritonavir and molnupinavir) were introduced in January 2022, further adding to the variability of therapeutic options and treatment regimens prescribed to study participants. However, on March 10, 2023, the Italian Medicines Agency banned the use of molnupinavir following recommendations from the European Medicine Agency (EMA), which cited insufficient clinical evidence regarding its effectiveness in reducing mortality and hospitalization rates. Furthermore, our analysis was conducted on a relatively small sample of patients.

## 5 Conclusions

Our results, in accordance with the literature, highlight the complexity of COVID-19 management in oncohematological patients, who are still at high risk of progression and death.

Even if early therapy may fail to prevent hospitalization and COVID-19 pneumonia in oncohematological patients, it still appears to be effective in reducing mortality. Pharmacological treatment combining different anti-SARS-CoV-2 drugs should be tailored to improve prognosis and viral clearance in patients with prolonged or relapsing infections.

Moreover, oncohematological patients with COVID-19 constitute a heterogeneous population with diverse clinical characteristics. Further studies with larger sample sizes may be needed to confirm our results and assess their clinical relevance.

## Data Availability

The original contributions presented in the study are included in the article/**Supplementary Material**. Further inquiries can be directed to the corresponding author.
