# Supplementary material for: Effectiveness of early intervention and combination treatment with monoclonal antibodies and antivirals in oncohematological patients with SARS-CoV-2: a retrospective experience
Source: Front Immunol. 2025 Mar 28;16:1524525. doi: 10.3389/fimmu.2025.1524525 (PMC11985841; doi:10.3389/fimmu.2025.1524525)
Supplement: Supplementary file 1 [file Table1.pdf]

Table 1: Adverse reaction to anti-SARS-CoV-2 treatment options in the overall population.

**Different drugs**	**Overall administrations**	**Adverse drug reaction n (%)**	**Type of drug reaction n (%)**	**Severity of drug reaction**
**Remdesivir**, distributed in:<br><br>Early therapy n=7<br>Monotherapy n=34<br>Association therapy n=43<br>Triple n=7	Global administrations n =91	3 (3.29)	Hypertransaminasemia = 3 (3.29)	Mild
**MoAb**, distributed in:<br><br>Early therapy n=4<br>Monotherapy n=9<br>Association therapy n =43<br>Triple n=7	Global administrations n=59	0 (0)	0 (0)	-
**Nirmatrelvir-ritonavir**, distributed in:<br><br>Early therapy n =6<br>Triple n=7	Global administrations n=13	4 (3.07)	Dysgeusia =3 (23.07)<br>Diarrhea =1 (7.69)	Mild<br>Moderate
**Molnupinavir**, distributed in:<br><br>Early therapy n=4	Global administrations n=4	0 (0)	0 (0)	-

Abbreviations: MoAbs, monoclonal antibodies; triple therapy (two antivarls+ one MoAb)
